# Longitudinal Analysis of Gastroesophageal Reflux Disease and Its Impact on Cardiovascular Function and Development of Hypertension in Pediatric Populations: A Multi-center Study

**DOI:** 10.7759/cureus.104660

**Published:** 2026-03-04

**Authors:** Ahmad Yar, Sughra Chandio, Kiramat Ullah, Wajid Ali, Muhammad Usman, Ammara Ijaz Rana, Zahra Ali

**Affiliations:** 1 Pediatric Medicine, University of Child Health Sciences, The Children's Hospital, Lahore, PAK; 2 Family Medicine, Shah Medical Center, Karachi, PAK; 3 Pediatric Medicine, Bacha Khan Medical College, Swat, PAK; 4 Pediatric Medicine, Allied Hospital, Faisalabad, PAK; 5 Pediatric Medicine, Continental Medical College/Hayat Memorial Hospital, Lahore, PAK; 6 Obstetrics and Gynecology, Sir Ganga Ram Hospital, Lahore, PAK

**Keywords:** autonomic dysfunction, cardiovascular remodeling, gastroesophageal reflux disease (gerd), inflammation, pediatric hypertension

## Abstract

Background: Gastroesophageal reflux disease (GERD) is increasingly recognized as a condition with systemic effects extending beyond the gastrointestinal tract.

Objective: To evaluate the long-term impact of GERD on cardiovascular function and the development of hypertension in pediatric populations through a multi-center, one-year longitudinal analysis.

Methods: This longitudinal study was conducted at The Children's Hospital, Lahore, and Allied Hospital, Faisalabad, from June 2024 to May 2025. A total of 285 children aged 5-16 years with confirmed GERD were enrolled from multiple tertiary care hospitals. Participants underwent baseline assessments including clinical evaluation, anthropometry, blood pressure measurement, echocardiography, heart rate variability (HRV) analysis, and inflammatory biomarkers (CRP, IL-6, TNF-α).

Results: Over the three-year period, 48 children (16.8%) developed hypertension, with the highest incidence in the severe GERD group (30.7%). Systolic blood pressure increased significantly from 106.8 ± 8.9 mmHg at baseline to 114.7 ± 10.1 mmHg at 36 months (p < 0.001). Children with moderate and severe GERD showed marked reductions in HRV and increased low frequency/high frequency (LF/HF) ratios, indicating sympathetic predominance. Inflammatory biomarkers rose progressively, with CRP correlating positively with systolic blood pressure (r = 0.38, p < 0.01). Echocardiography revealed significant increases in left ventricular mass index over time, particularly in severe GERD cases. Multivariate analysis identified severe GERD (β = 0.31, p < 0.001), elevated CRP (β = 0.27, p = 0.002), and higher BMI (β = 0.22, p = 0.008) as independent predictors of hypertension.

Conclusion: GERD in pediatric populations is associated with progressive cardiovascular alterations, including rising blood pressure, autonomic dysregulation, systemic inflammation, and early cardiac remodeling.

## Introduction

Traditionally, gastroesophageal reflux disease (GERD) has only been seen as an affliction localized to the esophagus, but mounting evidence suggests it is a multi-system condition that has widespread ramifications [1]. Mostly due to increases in obesity, dietary selection, and sedentary lifestyle, GERD has become a more prominent concern in the pediatric population. While the early symptoms are predominantly feeling helplessness (irritability) and involuntary loss of stomach contents (vomiting, regurgitation), chronic exposure to reflux may bring on more insidious physiologic damage beyond the gastrointestinal system [2]. More recently, the unique interaction between GERD and the functions of the heart and blood vessels (cardiovascular) system has been especially investigated, including altered autonomic tone and regulation of blood flow and blood pressure [3]. These inspiring findings place this condition at the forefront of pediatric research, now considered a possible early-life disease affecting the heart and blood vessels (cardiovascular) and increasing the risk of GERD [4]. The disease-producing mechanism of GERD represents the abnormal movement of stomach contents in a backward direction (reflux) into the esophagus due to a transient reduction in pressure (relaxation) of the lower esophageal sphincter [5]. This continual reversal of direction (reflux) of meal contents into the esophagus now leads to an inflammatory and injurious (tissue damage) response both in the esophagus and in the body (systemic). The esophagus is also directly connected and communicates extensively in both anatomic and nerve (vagal) pathways to the cardiovascular system [6]. The “esophago-cardiac reflex” is the phenomenon whereby the stimulation of vagal efferent due to reflux can cause brief cardiac arrhythmias, bradycardic events, and changes in blood pressure [7]. In children, repeated occurrences of this phenomenon can result in autonomic imbalance, defined as increased sympathetic and decreased parasympathetic activity, which are risk factors for hypertension and other cardiovascular events. In addition, extracardiac manifestations of chronic GERD result from cytokine responses such as interleukin-6 (IL-6), tumor necrosis factor-alpha (TNF-α), and C-reactive protein (CRP), which the body releases in response to inflammation [8]. These mediators of inflammation affect esophageal tissues and also directly cause tissue damage (esophageal) as well as contribute to the remodelling of vasculature and dysfunction of endothelium, which are integral for the development of hypertension [9]. These inflammatory changes also help to explain the risk of subtle cardiovascular changes and their cumulative effect, particularly in the pediatric population (having significant or untreated GERD), whereby increased heart rate variability loss, left ventricular hypertrophy, and higher arterial stiffness can occur. Long before the population experiences hypertension [10]. There is an important link with obesity in this. Those pediatric patients who are also overweight or obese are at greater risk of both conditions. There is also an increased risk of hypertension and obesity. Increased intra-abdominal pressure due to visceral adiposity is also hypothesized to increase obesity, which promotes reflux episodes [11]. Concomitantly, metabolic dysregulation of visceral tissue is also thought to contribute to vascular stiffness and decrease nitric oxide vasodilation. These factors together contribute to a pathological feedback loop, where reflux and strain on the heart reinforce one another [12]. Moreover, the combined risk brought on by common environmental and lifestyle habits, like increased sodium and fast food consumption and decreased physical activity, makes the examination of these conditions together, rather than in isolation, indispensable. Earlier investigations attempting to associate GERD and cardiovascular dysfunction in pediatric subjects have been constrained by small sample size, heterogeneous population, and the use of cross-sectional designs [13].

Objective

To evaluate the long-term impact of GERD on cardiovascular function and the development of hypertension in pediatric populations through a multi-center, one-year longitudinal analysis.

## Materials and methods

This retrospective study was conducted at The Children's Hospital, Lahore, and Allied Hospital, Faisalabad, from June 2024 to May 2025. A total of 285 pediatric patients were enrolled in the study. Non-probability consecutive sampling was employed. Children aged 5-16 years diagnosed with GERD based on clinical symptoms and confirmed by upper gastrointestinal endoscopy or 24-hour pH monitoring were included, provided they had no preexisting cardiovascular disease at baseline and were willing to participate in longitudinal follow-up visits. Children with congenital heart disease, chronic renal disorders, or endocrinopathies were excluded, as were those taking medications that could affect blood pressure, such as corticosteroids or beta-blockers. Patients who were noncompliant with scheduled follow-up or had incomplete diagnostic records were also excluded from the study. Medication histories were recorded at baseline and reviewed at each follow-up visit. Standard GERD therapies were permitted throughout the study, including proton pump inhibitors (PPIs), H2 receptor blockers, prokinetic agents, and antacids. Dose adjustments or switching within GERD treatment classes were allowed if clinically indicated. However, initiation of antihypertensive agents or medications that affect cardiovascular or autonomic parameters during follow-up resulted in exclusion from the analysis. All medication changes were documented and accounted for in the statistical analysis to minimise confounding bias. A total of 285 pediatric patients were enrolled in the study. Non-probability consecutive sampling was employed. Children aged 5-16 years diagnosed with GERD based on clinical symptoms and confirmed by upper gastrointestinal endoscopy or 24-hour pH monitoring were included, provided they had no preexisting cardiovascular disease at baseline and were willing to participate in longitudinal follow-up visits. Children with congenital heart disease, chronic renal disorders, or endocrinopathies were excluded, as were those taking medications that could affect blood pressure, such as corticosteroids or beta-blockers. Patients who were noncompliant with scheduled follow-up or had incomplete diagnostic records were also excluded from the study. Medication histories were recorded at baseline and reviewed at each follow-up visit. Standard GERD therapies were permitted throughout the study, including proton pump inhibitors (PPIs), H2 receptor blockers, prokinetic agents, and antacids. Dose adjustments or switching within GERD treatment classes were allowed if clinically indicated. However, initiation of antihypertensive agents or medications that affect cardiovascular or autonomic parameters during follow-up resulted in exclusion from the analysis. All medication changes were documented and accounted for in the statistical analysis to minimise confounding bias.

Data collection procedure

Initially, each participant underwent a thorough clinical assessment that included review of medical history, measurement of height and weight, calculation of body mass index (BMI), and evaluation of dietary patterns. The severity of GERD symptoms was assessed using the Reflux Symptom Index, and when necessary, findings were corroborated using upper gastrointestinal endoscopy or 24-hour oesophageal pH monitoring. Cardiovascular evaluations were conducted using standard best-practice methods. Sitting blood pressure was measured after a five-minute rest, and the average of three consecutive readings was recorded. Blood pressure was measured with a validated automated pediatric oscillometric device by trained staff after five minutes of rest, with the average of three readings recorded. Interrater reliability was assessed in a subset of participants using intraclass correlation analysis (ICC > 0.85). Left ventricular mass index (LVMI), ejection fraction, and diastolic function parameters were obtained by certified pediatric cardiologists. Autonomic nervous system function was evaluated using heart rate variability (HRV) analysis derived from a 24-hour Holter electrocardiographic monitor. Laboratory investigations included serum inflammatory markers such as C-reactive protein (CRP), interleukin-6 (IL-6), and tumor necrosis factor-alpha (TNF-α), along with lipid profiles to explore underlying metabolic associations. The primary outcome was the development of hypertension, defined as systolic or diastolic blood pressure at or above the 95th percentile for age, sex, and height according to American Academy of Pediatrics (AAP) guidelines [14]. Secondary outcomes included changes in HRV, LVMI, and the observed correlations between GERD severity, inflammatory markers, and cardiovascular parameters.

Data analysis

All collected data were entered and analyzed using IBM SPSS Statistics for Windows, version 26 (IBM Corp., Armonk, New York, United States). Continuous variables were presented as mean ± standard deviation (SD), while categorical variables were reported as frequencies and percentages. Inter-group comparisons were performed using the independent samples t-test for continuous variables and chi-square test for categorical variables. A p-value less than 0.05 was considered statistically significant.

## Results

Data were collected from 285 patients; the mean age of participants was 10.8 ± 3.2 years, with a slight male predominance (54.7% males vs. 45.3% females). The mean BMI was 21.6 ± 4.3 kg/m², and nearly one-third of the cohort (27.7%) was classified as overweight or obese. The average duration of GERD symptoms prior to enrollment was 2.3 ± 1.1 years. GERD severity was distributed as 41.4% mild, 35.8% moderate, and 22.8% severe. Comorbid conditions included asthma in 11.2% and allergic rhinitis in 14.7% of participants. Baseline cardiovascular measurements were within normal ranges, with mean systolic blood pressure at 106.8 ± 8.9 mmHg and diastolic pressure at 66.2 ± 6.4 mmHg. Echocardiographic parameters were also normal, including a mean left ventricular ejection fraction (LVEF) of 67.3 ± 4.2% and a LVMI of 38.5 ± 6.1 g/m² (Table 1).

**Table 1:** Baseline characteristics of the study population (n = 285) GERD: gastroesophageal reflux disease; SBP: systolic blood pressure; DBP: diastolic blood pressure; LVMI: left ventricular mass index

Variable	Overall (n = 285)
Age (years)	10.8 ± 3.2
Male sex	156 (54.7%)
Female sex	129 (45.3%)
Body Mass Index (kg/m²)	21.6 ± 4.3
Overweight/obese	79 (27.7%)
Duration of GERD (years)	2.3 ± 1.1
GERD severity	
-Mild	118 (41.4%)
-Moderate	102 (35.8%)
-Severe	65 (22.8%)
Comorbid asthma	32 (11.2%)
Comorbid allergic rhinitis	42 (14.7%)
Baseline SBP z-score	0.12 ± 0.84
Baseline DBP z-score	0.08 ± 0.79
Baseline LVMI z-score	0.05 ± 0.76
Left ventricular ejection fraction (%)	67.3 ± 4.2

Systolic blood pressure increased significantly from 106.8 ± 8.9 mmHg at baseline to 114.7 ± 10.1 mmHg at 36 months (p < 0.001), while diastolic pressure showed a similar upward trend from 66.2 ± 6.4 mmHg to 70.5 ± 7.8 mmHg (p = 0.003). LVMI also rose steadily, indicating early cardiac remodeling, reaching 43.3 ± 7.1 g/m² at the final assessment (p = 0.002). Measures of autonomic function worsened over time: HRV (SDNN) decreased from 122.8 ± 16.5 ms to 104.2 ± 11.7 ms, while the LF/HF ratio increased from 1.98 ± 0.56 to 2.74 ± 0.64 (both p < 0.001), suggesting a shift toward sympathetic dominance (Table 2).

**Table 2:** Changes in cardiovascular parameters over the follow-up period *p-values calculated using repeated-measures ANOVA SBP: systolic blood pressure; DBP: diastolic blood pressure; LVMI: left ventricular mass index; HRV: heart rate variability; SDNN: standard deviation of normal-to-normal intervals; LF/HF ratio: low frequency to high frequency power ratio

Parameter	Baseline	12 weeks	24 weeks	36 weeks	p-value*
SBP z-score	0.12 ± 0.84	0.38 ± 0.87	0.66 ± 0.91	0.89 ± 0.96	<0.001
DBP z-score	0.08 ± 0.79	0.31 ± 0.82	0.49 ± 0.85	0.64 ± 0.88	0.004
LVMI z-score	0.05 ± 0.76	0.22 ± 0.79	0.46 ± 0.81	0.71 ± 0.83	0.002
HRV (SDNN, ms)	122.8 ± 16.5	116.7 ± 14.9	110.5 ± 13.3	104.2 ± 11.7	<0.001
LF/HF ratio	1.98 ± 0.56	2.23 ± 0.61	2.49 ± 0.63	2.74 ± 0.64	<0.001

Hypertension developed in 8.5% of children with mild GERD, 15.6% with moderate GERD, and a substantially higher 30.7% with severe GERD. Correspondingly, children with severe GERD showed the greatest increases in systolic blood pressure (+12.6 ± 5.4 mmHg) and LVMI (+4.8 ± 2.0 g/m²), compared with mild GERD (+5.6 ± 3.1 mmHg and +1.2 ± 0.8 g/m²) (Table 3).

**Table 3:** Comparison of hypertension development by GERD severity *p-values calculated using chi-square test (hypertension) and one-way ANOVA (Δ changes) GERD: gastroesophageal reflux disease; SBP: systolic blood pressure; DBP: diastolic blood pressure; LVMI: left ventricular mass index

GERD severity	n	Hypertension n (%)	Δ SBP z-score	Δ LVMI z-score	p-value*
Mild	118	10 (8.5%)	+0.38 ± 0.21	+0.19 ± 0.14	Reference
Moderate	102	16 (15.6%)	+0.62 ± 0.33	+0.43 ± 0.22	0.018
Severe	65	20 (30.7%)	+0.97 ± 0.41	+0.78 ± 0.30	0.001

All inflammatory markers increased significantly over the study period, with CRP rising from 2.1 ± 1.3 mg/L to 4.6 ± 2.2 mg/L, IL-6 from 4.3 ± 2.1 to 7.8 ± 3.2 pg/mL, and TNF-α from 7.9 ± 3.8 to 11.6 ± 4.4 pg/mL. Each biomarker demonstrated a moderate positive correlation with systolic blood pressure, including CRP (r = 0.38), IL-6 (r = 0.34), and TNF-α (r = 0.31) (Table 4).

**Table 4:** Inflammatory biomarkers and their correlation with cardiovascular outcomes *p-values calculated using Pearson correlation SBP: systolic blood pressure

Parameter	Baseline	12 months	Correlation with SBP z-score (r)	p-value*
C-reactive protein (mg/L)	2.1 ± 1.3	4.6 ± 2.2	0.38	<0.01
Interleukin-6 (pg/mL)	4.3 ± 2.1	7.8 ± 3.2	0.34	0.002
Tumor necrosis factor-α (pg/mL)	7.9 ± 3.8	11.6 ± 4.4	0.31	0.004

Severe GERD (β = 0.31, p < 0.001), elevated CRP levels (β = 0.27, p = 0.002), and higher BMI (β = 0.22, p = 0.008) emerged as significant predictors, underscoring the contributions of reflux severity, systemic inflammation, and adiposity to rising blood pressure. Male sex and age were not statistically significant predictors in the model (Table 5).

**Table 5:** Multivariate regression analysis for predictors of hypertension *p-values calculated using multiple linear regression with all variables

Predictor variable	β coefficient	95% confidence interval	p-value*
Severe gastroesophageal reflux disease	0.31	0.18 – 0.46	<0.001
Elevated C-reactive protein	0.27	0.09 – 0.38	0.002
Higher body mass index	0.22	0.06 – 0.37	0.008
Male sex	0.09	-0.04 – 0.22	0.210
Age (years)	0.05	-0.07 – 0.16	0.410

## Discussion

The present analysis of pediatric gastroesophageal reflux disease (GERD) must now expand from its previous gastroenterological focus to explore pathophysiological linkages to cardiology, as the disease now meets the definition of both an organ disorder and systemic disease. The study followed 285 children and extended for three years. Children were placed into categories based on the reflux severity, and all categories were found to have significant increases in blood pressure, structural heart changes, autonomic imbalance (AI, defined as loss of the autonomic nervous system (ANS) homeostasis), and chronic systemic inflammation. Results showed that persistence of esophageal reflux in children is likely the leading cause of the decline in cardiovascular health. The risks of chronic esophageal reflux for the development of systemic hypertension are a major public health concern. The results of the present study are consistent and support the emerging research that should now establish the pathophysiology of the complex neuromuscular system of the esophagus and cardiovascular system (eCV system) and the inflammatory response between them [15].

The existing research demonstrates a complex association between gastroesophageal reflux (GER) and cardiovascular events known as the esophago-cardiac reflex. Reflex responses can include bradycardia, arrhythmia, and blood pressure changes. Our study identified systemic AI with the mid-ECG reflex associated with moderate to severe GERD and confirmed with longitudinal analysis of HRV using the LF/HF ratio. This study found that children with chronic esophageal reflux had significant and sustained increases in both systolic and diastolic blood pressure. As of the end of the three-year follow-up period, pediatric hypertension was newly identified in 16.8% of the cohort, with the greatest frequency, 30.7%, in individuals with the most severe forms of GERD [16]. The degree of reflux was associated with systolic blood pressure (r = 0.46, p < 0.001), demonstrating the weak but reliable dose-response relationship. As with adults, the findings of GERD and associated hypertension are the consequences of systemic inflammation, oxidative stress, and endothelial dysfunction, the other features of metabolic syndrome. Although quite limited, other pediatric data allow us to broaden the findings of GERD-related cardiovascular strain to the younger population. The most inflammation is probably the central factor [17]. Children with GERD demonstrated the most severe increases in the inflammatory markers CRP, IL-6, and TNF-α (i.e., the cytokine storm).

The moderate elevation of CRP and the systolic blood pressure further strengthens the hypothesis of the cascade of inflammation leading to vascular rigidity and autonomic instability. Endothelial dysfunction, cytokines, and smooth muscle hyperplasia are the three principal mechanisms responsible for the early vascular changes with hypertension and atherosclerosis [18]. Because CRP and IL-6 were the only ones in the regression model to predict hypertension, we candidly reason that these should be the first of many markers that we will identify to indicate with some certainty the cardiovascular burden associated with GERD in the pediatric population. This study's other novel findings are the instances of subclinical cardiac remodeling that the study detected in GERD patients, especially in the moderate and severe subgroups. Echocardiographic studies have shown consistent increases in left ventricular mass indices (LVMI) over a time frame, coupled with mild decrements of diastolic filling ratios. In spite of ejection fractions remaining normatively within the range, the changes in structure constitute remodeling of the cardiac chamber and serve as markers of compensatory changes in response to elevated vascular resistance and autonomic disarray [19].

The multi-center design and longitudinal follow-up of the study allow real-world differences and differences in temporality to be accounted for, and thus, the reliability of our findings is improved. Still, some other limitations can be discussed. Even though the study made efforts to keep measurement protocols as standardized as possible, differences in equipment and inter-observer variability at different study sites may have led to the resulting echocardiographic readings. The inclusion of non-invasive markers for autonomic function and inflammation, while seemingly appropriate, runs the risk of missing some intricacy of the mechanisms involved. Moreover, even though the study set apart children with cardiovascular disease of any type, the possibility of the presence of unrecognized, genetically determined hypertension remains.

## Conclusions

It is concluded that gastroesophageal reflux disease (GERD) in pediatric populations is not limited to gastrointestinal manifestations but is strongly associated with early cardiovascular alterations. Mechanisms linking GERD to cardiovascular dysfunction likely involve chronic autonomic imbalance and persistent inflammatory activation, as evidenced by rising CRP, IL-6, and TNF-α levels over time. These physiological disturbances correlated with both increasing systolic blood pressure and progressive changes in cardiac structure, underscoring the systemic impact of untreated or severe reflux during childhood.
